# Characterising a stress-sensitive default mode network (DMN) deficit in major psychiatric disorders

**DOI:** 10.1038/s42003-025-09400-1

**Published:** 2026-02-25

**Authors:** Sinead King, Zuo Zhang, Lauren Robinson, Robert Whelan, Frauke Nees, Marina Bobou, Tobias Banaschewski, Gareth J. Barker, Arun L.W. Bokde, Herta Flor, Antoine Grigis, Hugh Garavan, Penny Gowland, Andreas Heinz, Rüdiger Brühl, Jean-Luc Martinot, Marie-Laure Paillère Martinot, Eric Artiges, Luise Poustka, Sarah Hohmann, Nathalie Holz, Christian Baeuchl, Michael N. Smolka, Nilakshi Vaidya, Henrik Walter, Jeanne Winterer, M. John Broulidakis, Betteke Maria van Noort, Argyris Stringaris, Yuning Zhang, Julia Sinclair, Gunter Schumann, Ulrike Schmidt, Sylvane Desrivières, David Cotter, Allan H. Young, Gary Donohoe, Eric Wolztynski

**Affiliations:** 1 Futureneuro Research Ireland Centre for Translational Brain Science, Royal College of Surgeons, Dublin, Ireland; 2 Department of Psychological Medicine, Centre for Research in Eating and Weight Disorders, Institute of Psychiatry, Psychology & Neuroscience, King's College London, UK; 3 South London and Maudsley NHS Foundation Trust, London, UK; 4 Department of Psychological Medicine, Centre for Affective Disorders, Institute of Psychiatry, Psychology & Neuroscience, King's College London, 103 Denmark Hill, London, SE5 8AZ, UK; 5 School of Psychology and Global Brain Health Institute, Trinity College Dublin, Ireland; 6 Department of Child and Adolescent Psychiatry and Psychotherapy, Central Institute of Mental Health, Medical Faculty Mannheim, Heidelberg University, Square J5, 68159, Mannheim, Germany; 7 Institute of Cognitive and Clinical Neuroscience, Central Institute of Mental Health, Medical Faculty Mannheim, Heidelberg University, Square J5, Mannheim, Germany; 8 Institute of Medical Psychology and Medical Sociology, University Medical Center Schleswig-Holstein, Kiel University, Kiel, Germany; 9 University of Chinese Academy of Sciences, 100190 Beijing, China; 10 Brainnetome Center, Institute of Automation, Chinese Academy of Sciences, 100190 Beijing, China; 11 National Laboratory of Pattern Recognition, Institute of Automation, Chinese Academy of Sciences, 100190 Beijing, China; 12 Research Department of Clinical, Educational and Health Psychology, University College London, London, United Kingdom; 13 Department of Statistics, School of Mathematical Sciences, University College Cork, Cork, Ireland; 14 Department of Neuroimaging, Institute of Psychiatry, Psychology & Neuroscience, King’s College London, UK; 15 Discipline of Psychiatry, School of Medicine and Trinity College Institute of Neuroscience, Trinity College Dublin, Dublin, Ireland; 16 Department of Psychology, School of Social Sciences, University of Mannheim, 68131 Mannheim, Germany; 17 NeuroSpin, CEA, Université Paris-Saclay, F-91191 Gif-sur-Yvette, France; 18 Departments of Psychiatry and Psychology, University of Vermont, 05405 Burlington, Vermont, USA; 19 Sir Peter Mansfield Imaging Centre School of Physics and Astronomy, University of Nottingham, University Park, Nottingham, United Kingdom; 20 Charité – Universitätsmedizin Berlin, corporate member of Freie Universität Berlin, Humboldt-Universität zu Berlin, and Berlin Institute of Health, Department of Psychiatry and Psychotherapy, Campus Charité Mitte, Charitéplatz 1, Berlin, Germany; 21 Physikalisch-Technische Bundesanstalt (PTB), Braunschweig and Berlin, Germany; 22 Institut National de la Santé et de la Recherche Médicale, INSERM U1299 “Developmental trajectories & psychiatry”; Université Paris-Saclay, Université Paris Cité, Ecole Normale supérieure Paris-Saclay, CNRS; Centre Borelli UMR9010; Gif-sur-Yvette, France; 23 AP-HP. Sorbonne Université, Department of Child and Adolescent Psychiatry, Pitié-Salpêtrière Hospital, Paris, France; 24 Institut National de la Santé et de la Recherche Médicale, INSERM U 1299 "Trajectoires développementales en psychiatrie"; Université Paris-Saclay, Ecole Normale supérieure Paris-Saclay, CNRS, Centre Borelli; Gif-sur-Yvette, France; 25 Etablissement Public de Santé (EPS) Barthélemy Durand, 91700 Sainte-Geneviève-des-Bois, France; 26 Institut des Maladies Neurodégénératives, UMR 5293, CNRS, CEA, Université de Bordeaux, 33076 Bordeaux, France; 27 Departments of Psychiatry and Neuroscience and Centre Hospitalier Universitaire Sainte-Justine, University of Montreal, Montreal, Quebec, Canada; 28 Departments of Psychology and Psychiatry, University of Toronto, Toronto, Ontario, Canada; 29 Department of Child and Adolescent Psychiatry and Psychotherapy, University Medical Centre Göttingen, von-Siebold-Str. 5, 37075, Göttingen, Germany; 30 Department of Psychiatry and Psychotherapy, Technische Universität Dresden, Dresden, Germany; 31 Centre for Population Neuroscience and Stratified Medicine (PONS), Department of Psychiatry and Neuroscience, Charité Universitätsmedizin Berlin, Germany; 32 Department of Education and Psychology, Freie Universität Berlin, Berlin, Germany; 33 Clinical and Experimental Sciences, Faculty of Medicine, University of Southampton, Southampton, UK; 34 Department of psychology, College of Science, Northeastern University, Boston, MA, USA; 35 Department of Psychology, MSB Medical School Berlin, Rüdesheimer Str. 50, 14197 Berlin, Germany; 36 Division of Psychiatry and Department of Clinical, Educational & Health Psychology, University College London, London, UK; 37 Psychology Department, B44 University Rd, University of Southampton, Southampton SO17 1PS, UK; 38 School of Medicine, Center for Neuroimaging, Cognition and Genomics, National University of Ireland (NUI) Galway, Ireland; 39 Centre for Population Neuroscience and Precision Medicine (PONS), Institute for Science and Technology of Brain-inspired Intelligence (ISTBI), Fudan University, Shanghai, China; 40 Social, Genetic and Developmental Psychiatry Centre, Institute of Psychiatry, Psychology & Neuroscience, King’s College London, De Crespigny Park, London, SE5 8AF, UK; 41 School of Psychology, University of Galway, Galway, Ireland; 42 Insight Centre for Data Analytics, University College Cork, Cork, Ireland

## Abstract

Childhood trauma (CT) is associated with cognitive impairment across major psychiatric disorders. We tested a novel transdiagnostic hypothesis that atypical connectivity of the default mode network (DMN) mediates the association between childhood trauma (CT) and cognitive impairment. The sample of 1851 individuals aged 18-25 included 433 patients with depression, eating disorders, alcohol use disorders, psychosis and ADHD) and were recruited as part of the ESTRA/STRATIFY/IMAGEN studies. CT was measured using the Childhood Trauma Questionnaire (CTQ). The CANTAB spatial working memory task was administered to assess cognition. Four *a priori* seeds of the default mode network (DMN) were measured during face processing, namely the medial prefrontal cortex (PFC), right lateral parietal (LP), left lateral parietal (LP) and posterior cingulate cortex (PCC), according to the Harvard-Oxford Cortical and Subcortical Atlas (http://www.cma.mgh.harvard.edu/fsl_atlas.html) as implemented in CONN.

Patients had significantly reduced DMN connectivity between the four chosen DMN seeds and the rest of the brain. Reduced DMN connectivity mediated the association between higher CT and worse cognitive performance. Our findings are transdiagnostic in nature with stronger effects in some regions observed in depression, and suggest one transdiagnostic cortical network via which CT’s effects on cognition are transmitted.

## Introduction

More than two decades ago, it was established that adverse childhood traumatic experiences (CT) (i.e., childhood emotional and physical neglect or abuse, and sexual abuse) substantially increase the risk of developing a severe psychiatric condition (Sadowski, Ugarte, Kolvin, Kaplan, & Barnes, 1999). Despite significant scientific advances aimed at understanding this relationship, however, children who have been exposed to CT still have poorer long term treatment outcomes compared to children who have not (Karsberg, Hesse, Pedersen, Charak, & Pedersen, 2021; Kilian et al., 2020; Nanni, Uher, & Danese, 2012), with around 20% of the world’s children and adolescents being diagnosed with a psychiatric condition, and suicide being the second leading cause of death among 15-29-year-olds (World Health Organisation, 2023). The mechanism via which CT leads to poor treatment outcomes is unclear, but it has been established that such trauma can profoundly alter brain development, impacting key structures like the prefrontal cortex, amygdala, and the hippocampus. Growing evidence suggests that CT experiences are strongly associated with deficits in cognition, across major psychiatric disorders (Rokita, Dauvermann, & Donohoe, 2018).

Cognition refers to a broad range of mental processes including *general deficits such as* attention, decision-making, problem solving, language, behavioural, thought, self-regulation and memory, and *social cognitive deficits such as* perceiving, responding to and interacting with social cues, mind wandering, theory of mind, and emotion recognition. Dysfunctions between and within these processes have wide-ranging correlates and are related to problems in general adjustment, emotional and social functioning, and well-being. In psychiatric disorders, cognitive functioning independently predicts long-term illness course and poor psychosocial functioning (Cowman et al., 2021; Etkin, Gyurak, & O’Hara, 2013; Fett et al., 2011), even after accounting for clinical symptoms like hallucinations in schizophrenia or mood regulatory problems and rumination in depression. This highlights the importance of addressing cognitive dysfunctions as a crucial aspect of comprehensive psychiatric treatment. Recognizing the profound impact of cognitive dysfunction on psychiatric disorders, researchers are increasingly focusing on understanding and therapeutically targeting cognitive impairments, i.e., identification of biological markers could lead to novel medications, cognitive training interventions, and neuromodulatory therapies tailored to specific cognitive deficits.

Understanding the mechanism via which CT impacts cognition is therefore important, and recent work highlights a role of brain network default mode network (DMN) connectivity, in a manner that may explain at least part of the neurodevelopmental pathway via which CT impacts cognition. The Default Mode Network (DMN) is a set of brain regions that are active during social cognitive processes such as mind-wandering, introspection, emotion recognition and face processing. The DMN can become dysregulated, meaning its activity might be too high, too low, or improperly connected with other brain networks, resulting in social cognitive difficulties such as recognising and processing others emotions, as well as general cognitive deficits such as difficulty shifting attention, and impaired task switching. Therefore, understanding these specific cognitive deficits (i.e., emotional recognition, and cognitive flexibility) are crucial when aiming to understand DMN dysregulation.

Investigating emotional recognition and cognitive flexibility further and in a sample of adult patients with chronic schizophrenia, schizoaffective disorder and controls, we recently identified that DMN dysconnectivity during resting state conditions mediated CT’s effect on deficits in emotion recognition (King et al., 2021). The DMN is a well-established neural network of functionally and structurally connected brain regions that typically exhibit deactivation during the performance of an externally oriented attention-demanding task and increased activation during a resting state task. Atypical activation/weaker suppression of the DMN during task performance have been reported in some studies (Dauvermann et al., 2021; King et al., 2021; Mothersill et al., 2017; Wise et al., 2017) across major psychiatric disorders and may represent an impairment in functioning, or inability to suppress internal social cognitive oriented mental processes and focus on the task at hand. Some studies show that the DMN is typically activated in regions important for social cognitive processes (Grosbras & Paus, 2006; Soch et al., 2016), and has been largely linked to self-referential thought, mind-wandering, internal-oriented cognition, and social cognitive performance (Menon, 2023). Given recent evidence regarding associations between CT, DMN and cognition in chronic psychosis, the present study investigates whether these same associations exist across multiple psychiatric disorders, in a large sample of young adults aged 18-25 with Major Depression, Alcohol Use Disorder, Psychosis, Eating Disorders and ADHD. We further aim to examine whether any observed aberrant DMN connectivity in patients may mediate the association between CT and general cognitive deficits.

The aims of this study are therefore:

(i) To examine the association between CT and cognitive performance across participants in the STRATIFY, ESTRA and IMAGEN studies. The hypothesis is that higher CT will be associated with worse cognitive performance.
(ii) To compare differences in DMN during social cognition between patients and age and sex matched controls. The hypothesis is that there will be atypical DMN connectivity in patients compared to controls.
(iii) To examine whether any observed atypical DMN connectivity during social cognition mediates the association between childhood trauma and cognitive deficits. The hypothesis is that aberrant DMN connectivity would mediate the association between a higher past history of childhood trauma and cognitive deficits.

## Methods

### Participants

Participants were assessed as part of the patient samples ESTRA and STRATIFY Consortium, and a large population-based sample from the IMAGEN Consortium (Mascarell Maričić et al., 2020; Quinlan et al., 2020) (see demographic Supplementary Table 1 for N in each group). See below for specific details on each consortium’s project recruitment, where each consortium was purposefully designed to have the exact same assessments, to enable comparability.

**STRATIFY Consortium:**

○ **Diagnostic Groups:** Major Depression (MDD - moderate to severe PHQ-9), Alcohol Use Disorder (AUD - AUDIT >15), ADHD, or Psychosis (ICD-10/DSM-V criteria).
○ **Age Range:** 18-25 years.
○ **Study Sites:** London, Southampton, and Berlin.
○ **Note:** The ADHD group was excluded from specific patient group analysis due to N<5 but included in general patient vs. control analyses.

**IMAGEN Consortium (Population-based sample):**

○ **Diagnostic Groups:** Healthy Controls (screened negative for all psychiatric diagnoses using MINI, version 5.0.0).
○ **Age Range:** 18-25 years (at the third follow-up).
○ **Study Sites:** Eight study sites in Europe including London and Berlin.
○ **Note:** This was a longitudinal neuroimaging and genetics study of adolescents.

**ESTRA Consortium:**

○ **Diagnostic Groups:** Eating Disorders (ED), specifically Anorexia Nervosa (AN) or Bulimia Nervosa (BN) (DSM-V criteria).
○ **Age Range:** 18-25 years.
○ **Study Site:** London.
○ **Sex:** All female participants.

Patients with major depression (MDD), alcohol use disorder (AUD), and Psychosis, and healthy controls aged 18-25 years, were recruited as part of the STRATIFY study from three study sites: London, Southampton, and Berlin. The population based sample consisted of IMAGEN - a longitudinal neuroimaging and genetics study of adolescents recruited from eight study sites in Europe, and participants were included from this IMAGEN study at the third follow-up, aged 18-25 years. Patients with eating disorders (ED) were recruited as part of the ESTRA study, in which all participants were female, aged 18-25 years, and recruited at the London study site. All ethical regulations relevant to human research participants were followed. STRATIFY/ESTRA was approved by the London – Westminster Research Ethics Committee and The IMAGEN study was approved by local ethics research committees at each research site: King’s College London, University of Nottingham, Trinity College Dublin, University of Heidelberg, Technische Universität Dresden, Commissariatà l’Energie Atomique et aux Energies Alternatives and University Medical Center. 17/LO/1552: Approved by London and Westminster Ethical Review Board, at Kings College London, with sponsorship from NHS Health Research Authority. Written consent was obtained from all the participants before participation and all ethical regulations relevant to human research participants were followed. All samples had the same exclusion criteria; (i) participants with brain injuries including stroke, tumours, epilepsy, neurodegenerative or other neurological disorders (ii) participants who were deaf or had significant hearing problems or a hearing aid that cannot be removed, blind or vision difficulties (correct near vision of 20/100 or worse in both eyes); (iii) participants with type I or type II diabetes or heavily medicated for serious illness other than for the mental health issues under investigation; (iv) participants who were pregnant or any possibility that they may have been pregnant; (v) participants with restricted mobility, including inability to lie flat for 1.5 hours.

## Measures

### MRI

#### fMRI Emotional face processing task (EFT)

The EFT was adapted from Grosbras et al. (Grosbras & Paus, 2006). Participants watched 18-second blocks of either a face movie (depicting anger or neutrality) or a control stimulus. Each face movie showed black and white video clips (200–500 ms) of male or female faces. Five blocks each of angry and neutral expressions were interleaved with nine blocks of the control stimulus. Each block contained eight trials of six face identities (three female). The same identities were used for the angry and neutral blocks. The control stimuli were black and white concentric circles that expanded and contracted at various speeds, roughly matching the contrast and motion characteristics of the face clips. Our study used the EFT task conditions of neutral and angry faces.

#### Image acquisition

fMRI data were acquired at eight IMAGEN assessment sites, and three STRATIFY sites with 3 T MRI scanners from different manufacturers (Siemens, Philips, General Electric, Bruker). The scanning variables were specifically chosen to be compatible with all scanners. The same scanning protocol was used at all sites. In brief, high-resolution T1-weighted 3D structural images were acquired for anatomical localization and coregistration with the functional time series. In addition, blood oxygen level-dependent (BOLD) functional images were acquired with a gradient-echo, echo-planar imaging sequence. For all tasks, each volume consisted of 40 slices aligned to the anterior commission–posterior commission (2.4-mm slice thickness, 1-mm gap). The echo time (30ms) was chosen to provide reliable imaging of the subcortical areas, and repetition time (TR) was 2,200 ms.

#### Task-based functional image preprocessing

Task-based fMRI data were first prepreprocessed using SPM8 (Statistical Parametric Mapping, http://www.fil.ion.ucl.ac.uk/spm). Spatial preprocessing included slice time correction to adjust for time differences due to multislice imaging acquisition, realignment to the first volume in line, nonlinearly warping to the MNI space (on the basis of a custom echo-planar imaging template (53 × 63 × 46 voxels) created from an average of the mean images from 400 adolescents), resampling at a resolution of 3 × 3 × 3 mm^3^ and smoothing with an isotropic Gaussian kernel of 5 mm full-width at half-maximum. Seed-based functional connectivity was run in CONN-fMRI Functional Connectivity toolbox (version 18a), using SPM12, to assess functional connectivity of four *a priori* seeds of the default mode network (DMN), namely the medial prefrontal cortex (PFC), right lateral parietal (LP), left lateral parietal (LP) and posterior cingulate cortex (PCC), according to the Harvard-Oxford Cortical and Subcortical Atlas (http://www.cma.mgh.harvard.edu/fsl_atlas.html) as implemented in CONN.

To limit the effects of head motion, scans underwent ‘motion scrubbing’. Anatomical component-based noise correction (aCompCor) was implemented in CONN, employing structural white matter (WM) and cerebrospinal fluid (CSF) masks to generate effective motion-reducing regressors. This method has been shown to be effective at reducing the effects of head movement and there was no significant difference in head motion between patients and controls. Additional head motion variance was addressed by including regressors from the 6 motion correction parameters and their temporal derivatives (GM, WM, CSF) during band-pass filtering (.01–.10 Hz).

#### Childhood Trauma Questionnaire

Childhood trauma experiences were retrospectively measured using the Childhood Trauma Questionnaire (CTQ) (Bernstein et al., 1994) that assesses five types of trauma, including physical abuse, physical neglect, emotional abuse, emotional neglect and sexual abuse. Each subscale includes five items, and individuals are asked to respond whether they had experienced the event on a Likert scale ranging from ‘1′ (‘never true’) to ‘5′ (‘very often true’). For this study, the Likert scale was rescaled to the following values: ‘0’ (‘never true’) to ‘4’ (‘very often true’). Hence, the rescaled total score of each subscale ranged from ‘0’ to ‘100’ with higher scores reflecting greater frequency. The CTQ has strong psychometric properties as demonstrated in both clinical and non-clinical samples (Scher, Stein, Asmundson, McCreary, & Forde, 2001).

#### Cognitive Performance

The Cambridge Neuropsychological Test Automated Battery (CANTAB) is a semiautomated computer interface for assessing cognitive function (Robbins et al., 1994). The following CANTAB cognitive tests were used in this study:

#### Spatial Working Memory

The Spatial Working Memory (SWM) test by CANTAB is a test that examines spatial working memory. For this task, an increasing number of boxes were presented on screen during the trials. The participant was instructed to search for tokens, opening the boxes by touching them, and advised not to return to a box that had already yielded a token. The analyzed measures were SWM-between errors (number of times the participant revisited a box where a token had previously been found) and SWM-strategy (number of times the participant started a new search by touching a different box). Lower scores suggest that the participant used the strategy of following a predetermined sequence by beginning with a certain box, and when a token was found, he/she returned to that box to start a new search.

#### Intra-Extra Dimensional (IED) Shift

The Intra-Extra Dimensional Set Shift (IED) task assesses the processes involved in categorising stimuli into sets (visual discrimination of shapes vs. lines), and responding flexibly (shifting attention) to changes in stimuli. The IED task requires participants to learn the rule and select the correct icon (a specific shape or line). The task builds in complexity as distractors are added and the rule changes. The rule changes are both intra-dimensional (e.g. shapes are still the relevant set, but a different shape is now correct) and extra-dimensional (e.g. shapes are no longer the relevant set, instead one of the line stimuli is now correct).

#### Cambridge Gambling Task (CGT)

The Cambridge Gambling Task (CGT) is a computerized neurocognitive assessment designed to measure decision-making and risk-taking behavior in situations where the probabilities of winning or losing are explicitly known, rather than learned through trial and error. There are a number of outcome measures in this task but the present study used two main outcome measures called deliberation time and risk adjustment, as described below:

**Deliberation Time** refers to the amount of time a participant takes to make their decision during the task. It’s often used as an indicator of cognitive processing speed and consideration, with longer times potentially suggesting more cautious or slower decision-making.

**Risk Adjustment** measures a participant’s ability to modify their betting behavior (risk-taking) in response to changes in the probability of winning. A higher risk adjustment score indicates that the individual effectively alters their bets based on the odds, suggesting a more rational and adaptive approach to risk. Conversely, a lower score might suggest insensitivity to the changing probabilities, or a tendency to bet consistently regardless of the odds.

### Statistics and Reproducibility

An analysis was performed within CONN software (Whitfield-Gabrieli & Nieto-Castanon, 2012) to examine differences in DMN network connectivity between patients and controls. Significant differences were reported between groups for all four seeds of the DMN when examining each seed at the *a priori* defined statistical threshold of Family-wise error (FWE) cluster-level *P*_FWE_ < .001 for both the cluster-level and height threshold. In the present study, we derived connectivity metrics from case-control differences and these correlation coefficients were brought forward for subsequent mediation analysis when examining DMN differences as potential mediators of the association between CT and cognitive performance.

The correlation coefficients representing case-control differences were extracted from the CONN software to R (R Core Team, 2022; URL https://www.R-project.org/). Within R, further analyses were performed to assess the association between cognitive measures and the childhood traumatic event scores across all participants, whilst controlling for confounding variables. To further assess mediation, we modelled the indirect effect of the association between CT and cognition through brain connectivity as a mediator in a univariate mediation analysis as described by Hayes, model 4 (Hayes, 2012). 52 tests were run in total, which were corrected for, using FDR.

### Predictors (Independent variable IV): Childhood Trauma

We used the CT total score as the main independent trauma variable (IV) when examining the association between CT and cognition. The CT total score variable was first used as our IV and for each subsequent analysis, we explored whether mediating effects would be observed using each other CT variable as our IV. To do this, we re-ran each bootstrap analysis i.e., using each of emotional abuse, physical abuse, sexual abuse, emotional neglect and physical neglect as IV separately. The univariate mediating effect and significance of each of these variables were respectively evaluated as the bootstrapped mean effect and p-value from 5000 resamples. The bootstrap framework was also used to obtain non-parametric 95% confidence intervals (CI) for effect sizes.

### Mediators

The significant DMN brain connectivity measures observed from the initial analysis were used as mediators.

### Outcomes (Dependent Variable DV): Cognitive function

The significant cognitive measures observed from the initial case-control analysis were taken further for mediation analysis and used as outcome / dependent variable measures.

All analysis were carried out across the entire sample. Moderated mediation analysis was carried out again, across the entire sample. Diagnosis was included as our moderator variable. Confounding variables included age, sex, IQ, BMI and site. Bootstrap CIs that did not include zero indicated a significant indirect effect (Hayes, 2012).

### Role of the funding source

The funding agencies did not play a role in any of the following activities: the study design; the collection, analysis, and interpretation of data; the writing of the report; and the decision to submit the paper for publication.

## Results

### Demographic, Clinical, Environmental, and Neuropsychological Data

Four hundred and thirty three patients and one thousand four hundred and eighteen controls took part in this study. Demographic, clinical, environmental, and neuropsychological data are presented in Supplementary Table 1. No significant differences between patients and controls were observed for either age (patients, mean = 21.7 years, SD ± 2.1; controls, mean = 21.9 years, SD ± 1.5; T = 1.5, *p* = .34) or sex (controls, 56% female; patients, 58% female; χ ^2^ = .81, *P* = .15).

### Childhood Trauma and Cognition

Patients scored significantly higher on all measures of the childhood trauma (CTQ) questionnaire compared to controls (p=<.001 on all subscales). Patients performed significantly worse compared to controls on all measures of neuropsychological functioning, i.e., on the Cambridge Gambling Task (CGT) Deliberation Time and Risk Adjustment scores (p=<.001 for both), the Intra-Extra Dimensional Shift (IED) Total Scores test (p=.021), and the spatial working memory (SWM) Strategy and Between Errors tasks (p=<.001 for both). A higher history of Total CTQ was associated with worse performance on both the SWM Between Errors (p=<.001) test and the SWM Strategy test (p=<.001). Further analyses were conducted to determine the magnitude of differences between the condition and control groups across all outcome measures, with effect sizes presented in Supplementary Table 1.

### Functional Connectivity of the Default-Mode Network in Patients Compared to Controls During Face Processing

**Figure 1:**
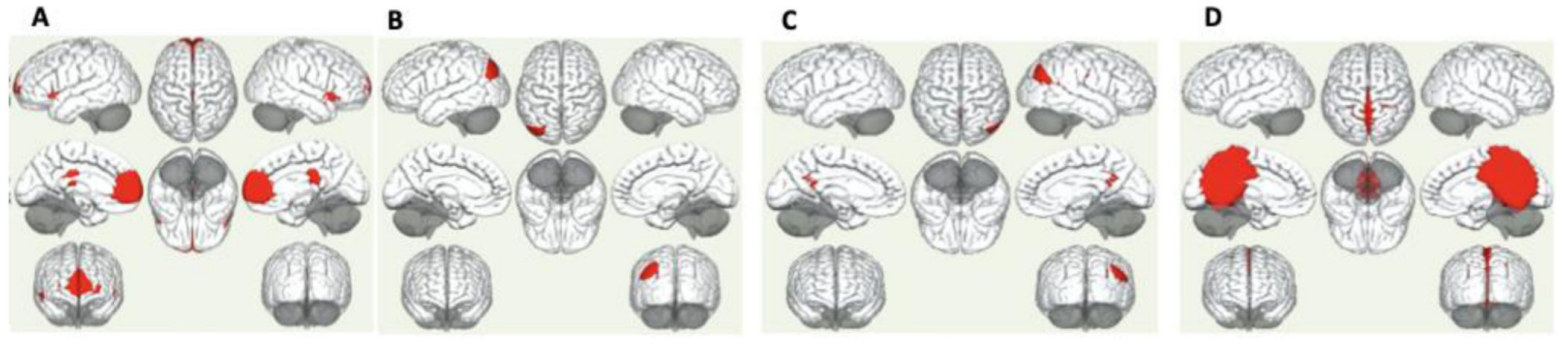
Differences in Functional Connectivity of the Default Mode Network between Patients and Controls

Seed-based functional connectivity between each of the four DMN seed regions chosen (see methods i.e., medial prefrontal cortex (mPFC), right lateral parietal (LP), left lateral parietal (LP), and the posterior cingulate cortex (PCC)) and the rest of the brain are shown for patients and controls in Figure 1. Comparisons between patients and controls showed significant differences between groups for all four seeds of the DMN (Supplementary Table 2). Differences between groups when examining each seed at the *a priori* defined statistical threshold of cluster-level *p*_FWE_ =< .001 for both the cluster-level and height threshold showed significantly stable clusters involving differences in DMN connectivity between groups.

**DMN Differences in medial prefrontal cortex (mPFC):** The mPFC showed significantly reduced connectivity in patients compared to controls, specifically in regions encompassing (1) the bilateral frontal pole and paracingulate gyrus (*p*_FWE_=<.001), (2) the posterior cingulate gyrus and thalamus (*p*_FWE_=<.001), (3) the left temporal pole and temporal gyrus (*p*_FWE_<.001), and (4) the right temporal pole and gyrus and right occipital cortex (*p*_FWE_=<.001). **DMN Differences in LLP:** When examining the left LP seed, we found differences between groups (decreased in patients) in regions encompassing (1) LLP and bilateral occipital cortex and precuneus (*p*_FWE_=<.001), (2) cingulate gyrus and precuneus <.001 (FWE), (3) left supramarginal gyrus (*p*_FWE_=<.001), and (4) a cluster encompassing another region of the precuneus (*p*_FWE_=<.001). **DMN Differences in RLP:** For the right LP seed, reduced functional connectivity differences between patients and controls were also observed in regions encompassing (1) right occipital cortex and right temporal gyrus (*p*_FWE_=<.001) (note precuneus was included in this cluster also), (2) the left angular gyrus, occipital cortex and precuneus (*p*_FWE_=<.001), (3) the cingulate gyrus, precuneus and thalamus (*p*_FWE_=<.001), and (4) the precuneus (*p*_FWE_=<.001). Note that clusters encompassing connectivity between RLP and the precuneus appear particularly relevant also. **DMN Differences in PCC:** Finally, functional connectivity differences were also observed between the PCC seed and a large cluster encompassing the precuneus and other brain regions (*p*_FWE_=<.001), which were reduced in patients compared to controls. A smaller cluster involving the right frontal gyrus was also observed in patients compared to controls (*p*_FWE_=<.001).

**Figure 2:**
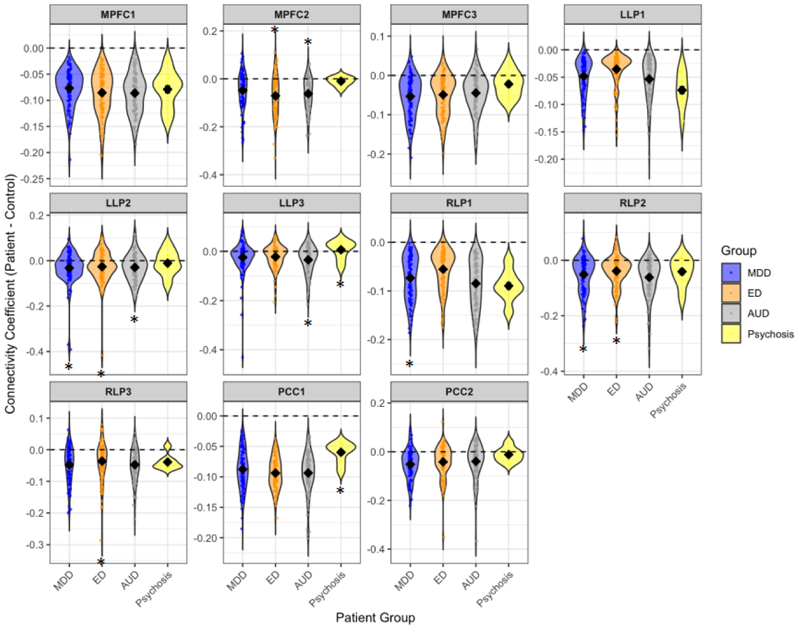
Differences in DMN connectivity between patient groups and controls **Note:** Patient group differences (MDD, ED, AUD, Psychosis) as compared to controls in functional connectivity of the default-mode network, where the y axis represents functional connectivity correlation coefficients (see methods). The bars represent coefficients of group differences and asterisks represent a statistically significant difference of <.05, where no asterisk means no significant difference was observed. See Supplemental Table 3 for individual data points (coefficients and standard error) presented here. Left to right: **the medial prefrontal cortex (mPFC)** and the **(mPFC1)** bilateral frontal pole and paracingulate gyrus **(mPFC2)** the posterior cingulate gyrus and thalamus **(mPFC3)** the left temporal pole and temporal gyrus; **left Lateral Parietal seed (LLP)** and the **(LLP1)** bilateral occipital cortex and precuneus, **(LLP2)** cingulate gyrus and precuneus, **(LLP3)** left supramarginal gyrus, **the right Lateral Parietal (RLP) seed** and the **(RLP1)** right occipital cortex and right temporal gyrus **(RLP2)** the left angular gyrus, occipital cortex and precuneus **(RLP3)** the cingulate gyrus, precuneus and thalamus**; the posterior cingulate cortex (PCC)** and **(PCC1)** a cluster encompassing the precuneus and **(PCC2)** posterior frontal gyrus. All results were corrected using FDR correction.

In terms of specific patient group differences, we compared DMN connectivity between each patient group and controls. We found that patients with eating disorders and patients with alcohol use disorders had significantly increased mPFC connectivity (specifically between the mPFC and posterior cingulate gyrus (PCC) and thalamus) compared to controls. Patients from all patient groups had reduced connectivity in specific regions of the left and right lateral cortices but not others and psychosis patients had reduced posterior cingulate cortex compared to controls. There were no other statistically significant differences (Figure 2).

### Greater reduction of the DMN during face processing mediates the association between higher CT exposure and Spatial Working Memory performance

We subsequently aimed to replicate the associations previously reported in King et al. (2021) through a comparable analysis. More specifically, we aimed to test whether the atypical DMN connectivity observed above in the present case versus control analysis would mediate the association between CT and cognitive impairment. We examined this using the spatial working memory test (SWM), i.e., with SWM subscale (i) SWM total errors and (ii) SWM strategy as our dependent variable (DV). We chose SWM as our DV because this variable was most strongly associated with CT in this dataset. We used the CT total score as the main independent trauma variable (IV). The CT total score variable was first used as our IV and DMN connectivity was used as our mediating (M) variable. For each subsequent analysis, we explored whether mediating effects would be observed using other CT variables as our IV. For this, we re-ran the bootstrap analyses i.e., for emotional abuse, physical abuse, sexual abuse, emotional neglect and physical neglect, successively.

**Figure 3:**
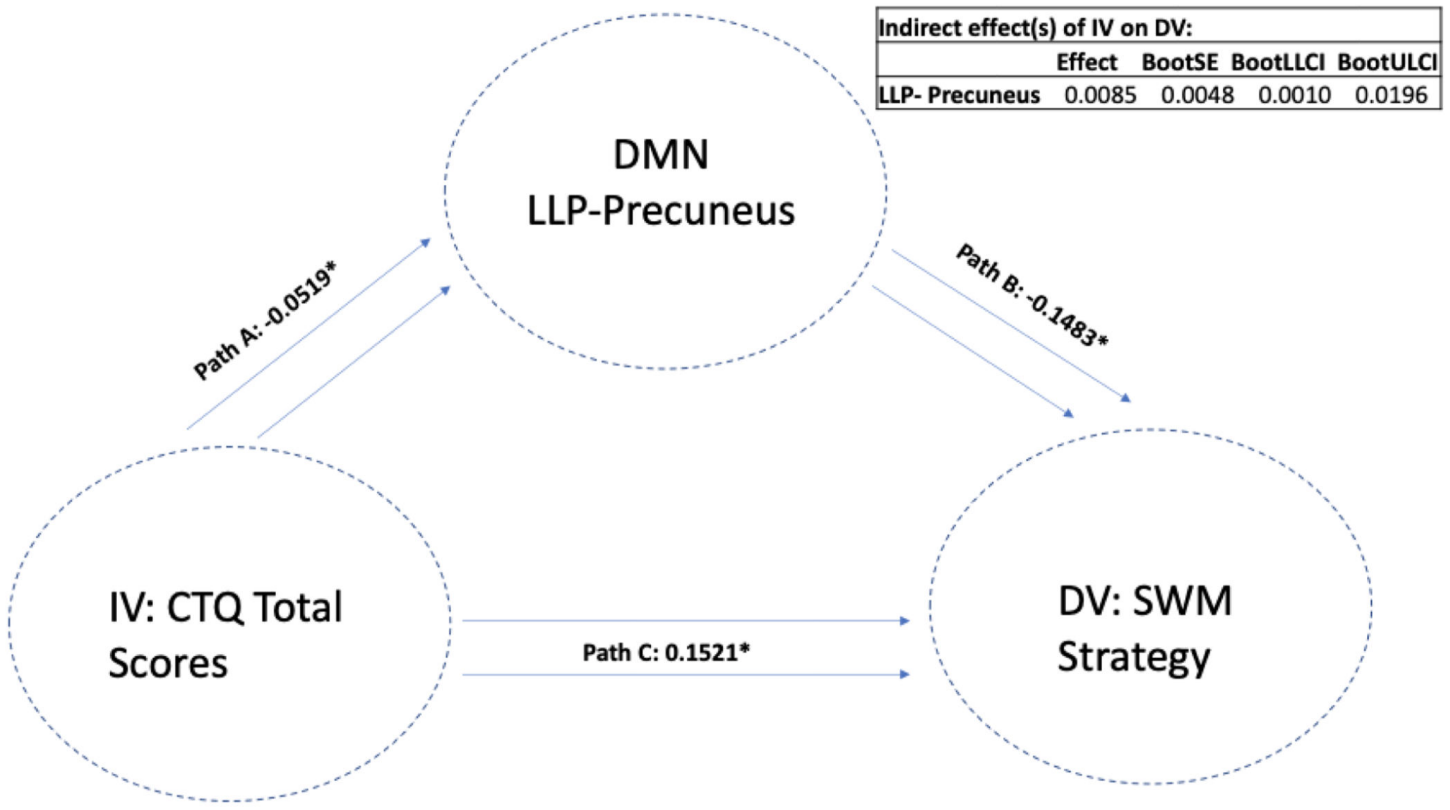
The mediating role of DMN-LLP on CTQ and SWM Strategy Note: Standardised effects are presented.

**Figure 4:**
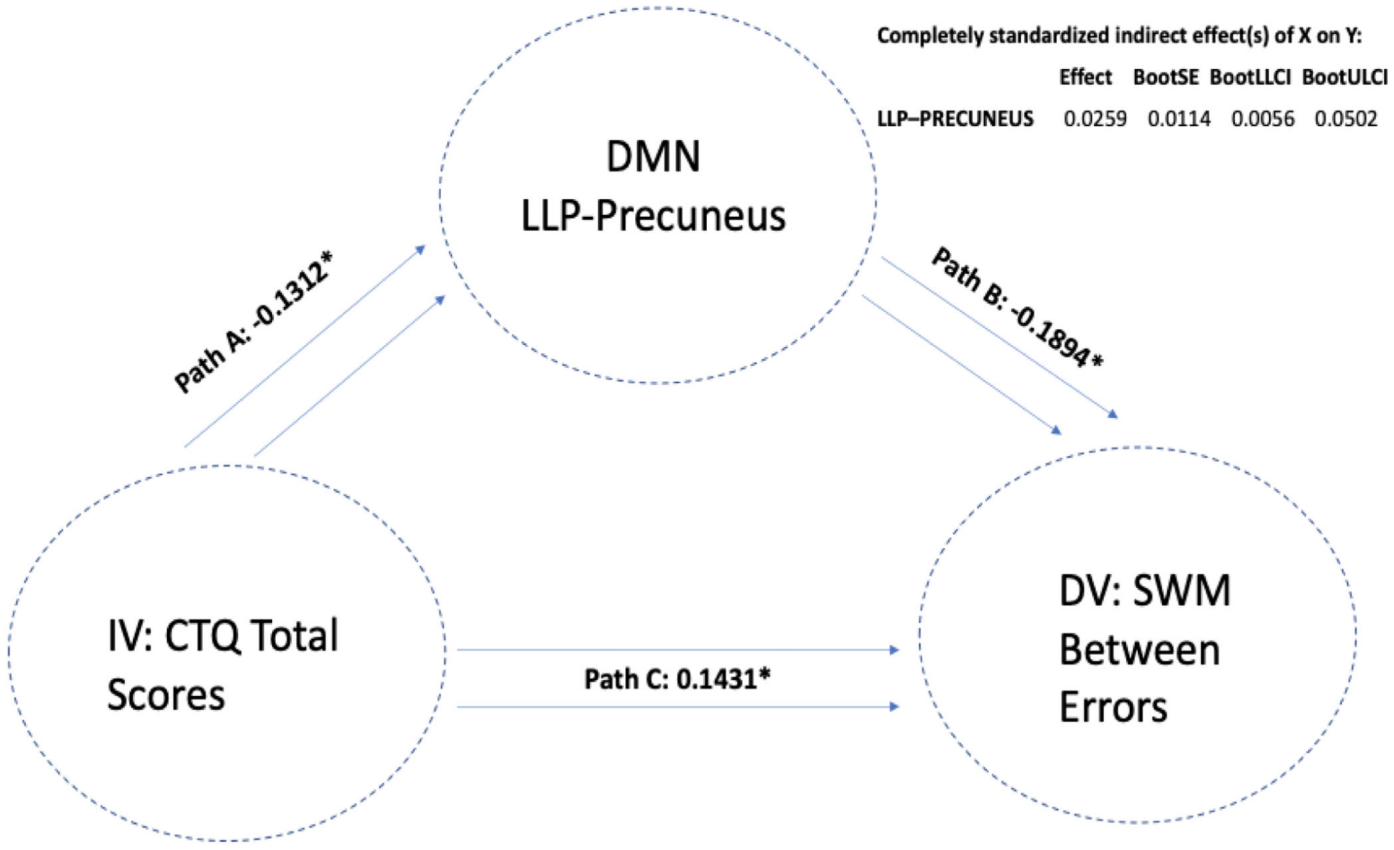
The mediating role of DMN-LLP on CTQ and SWM Between Errors Note: Standardised effects are presented.

**Figure 5:**
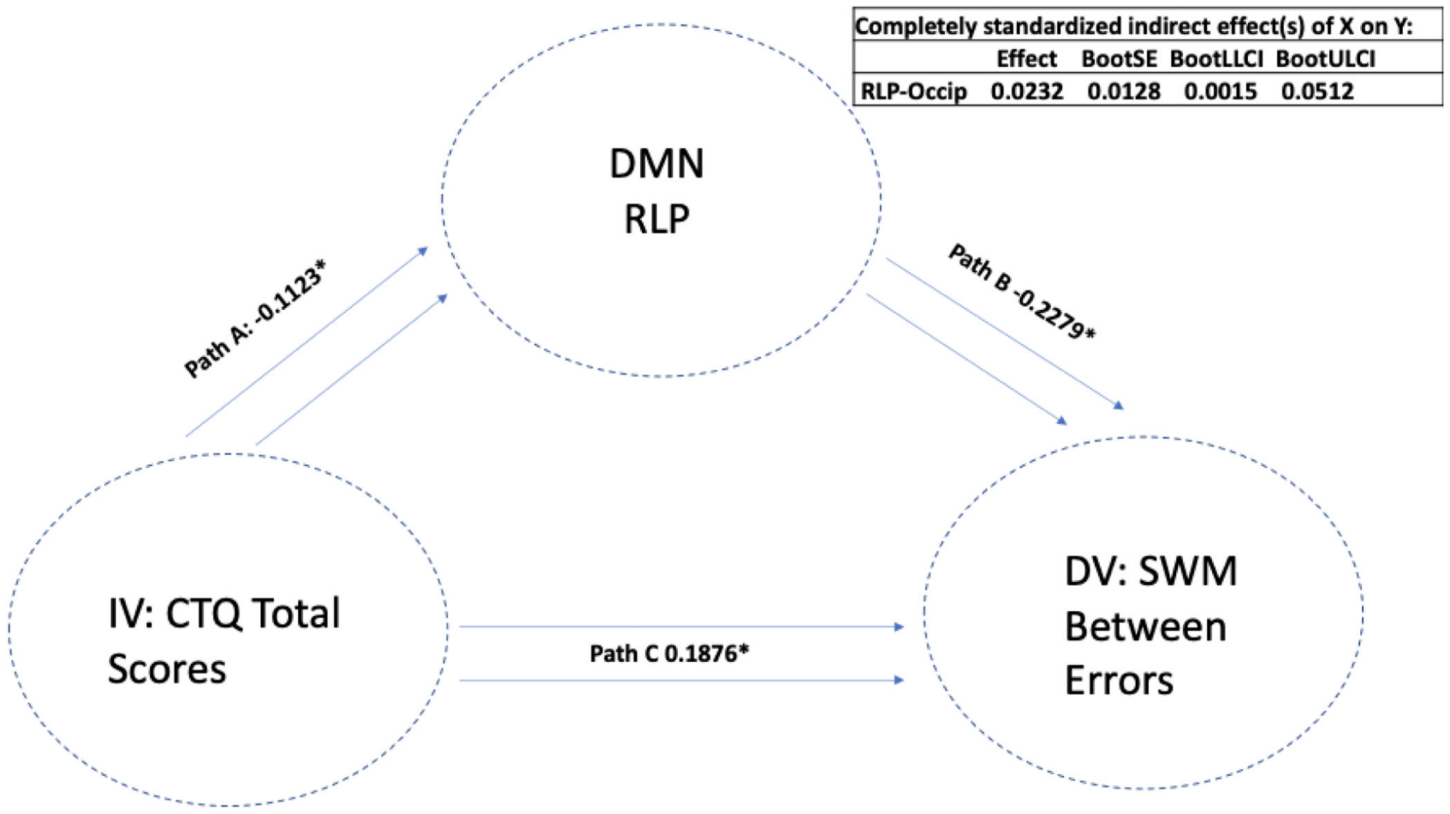
The mediating role of DMN-RLP on CTQ and SWM Between Errors Note: Standardised effects are presented.

There were significant indirect mediations found, where reduced DMN connectivity seed of the left lateral parietal cortex (with a cluster encompassing left occipital cortex and precuneus) mediated the association between a higher past history of childhood trauma and worse performance on both the spatial working memory total errors scale (LLP-Precuneus: p=.003, B= 0.0175, CI=(0.0021, 0.0390)) (i.e., a higher score means more errors) and the strategy scores (LLP-Precuneus: p=.004, B= 0.0175, CI=(0.0019, 0.0391)) (i.e., a higher score indicates a less effective strategy). This means that atypical DMN connectivity was found to mediate the association between higher CT and worse performance on SWM; see Figure 3. We next removed the CT total variable and replaced it with each CTQ sub-item to assess the importance of each sub-item scale. We again found that when examining the same LLP-precuneus seed, this seed mediated the association between a higher history of sexual abuse, emotional neglect and physical neglect (but not emotional and physical abuse) and worse performance on the SWM Strategy test. Next, we replaced SWM Strategy scores as DV with SWM Between Errors scores and re-ran the mediation model to examine whether the same LLP-precuneus variable mediated the association between CTQ-total scores and SWM Between Errors. We found that LLP-precuneus mediated the association between CT-total scores and SWM Between Errors; see Figure 4. We then replaced CTQ-total scores with other CTQ sub item scores and again found that this variable mediated the association between sexual abuse, emotional neglect and physical neglect (but not emotional or physical abuse) and SWM Between Error scores. We next replaced our mediating variable LLP-precuneus with other DMN seeds and re-ran the mediation analysis. Similar findings were observed with a DMN cluster encompassing right lateral parietal cortex (RLP) and right occipital cortex and precuneus, where RLP-precuneus mediated the association between CTQ-total, sexual abuse, emotional neglect and physical neglect (but not emotional and physical abuse) and SWM Between Error scores; see Figure 5. To examine whether this observed mediation was statistically moderated by patient group, we ran a moderated mediation model. We found that a diagnosis of major depression (MDD) significantly moderated the mediating effects observed (LLP-Precuneus: p=.012, B= -0.0007, p= 0.0002, CI=(-0.0011, -0.0002)), but only for the LLP-precuneus seed, meaning that the mediating effects observed were significantly stronger in patients with MDD when considering connectivity between LLP and precuneus. No other moderating effects were observed for other patient groups or when considering other variables.

## Discussion

In this study, we report significantly reduced connectivity of the default mode network (DMN) during social cognition in patients compared to controls. Further to this, we found that this observed atypically reduced DMN connectivity mediated the association between a higher past history of childhood trauma and worse performance on a spatial working memory (SWM) task. Childhood trauma is therefore associated with atypical DMN connectivity during a face processing task indicating abnormal social cognitive functioning, which in turn leads to (mediates) impairments in spatial working memory. These findings have important implications for treatment and understanding of mental health cognitive symptoms, evidence for which arises from one of the largest samples of young individuals suffering from psychiatric disorders.

The mPFC showed significantly reduced connectivity compared to controls, specifically in regions encompassing (1) the bilateral frontal pole and paracingulate gyrus (2) the posterior cingulate gyrus and thalamus (3) the left temporal pole and temporal gyrus and (4) the right temporal pole and gyrus and right occipital cortex. Differences in mPFC connectivity between patients and controls during task based conditions is in keeping with findings of some systematic reviews and meta-analyses (Anticevic et al., 2012) but not others (Scalabrini et al., 2020). The mPFC plays a key role in the social understanding of others, and the subregions of the mPFC contribute differently to this function according to their roles in different subsystems of the DMN (Li, Mai, & Liu, 2014). For example, the ventral mPFC in the medial temporal lobe (MTL) subsystem and its connections with emotion regions are mainly associated with emotion engagement during social interactions. The patients in this study had reduced connectivity between the mPFC and bilateral frontal pole and cingulate gyrus. Therefore, the results of the current study suggest that patients have less engagement of these mPFC-cingulate gyrus regions, functional connectivity that is likely important during social interactions and emotion recognition, as they are involved in regulating emotions and predicting negative consequences.

The anterior MPFC (aMPFC) in the cortical midline structures (CMS) and its connections with posterior cingulate cortex (PCC) contribute mostly to making self-other distinctions, and is considered pivotal in generating affective, self-directed judgements and thought (Wise et al., 2017). Interestingly, patients with eating disorders and alcohol use disorders also show hyperconnectivity of these connections between the mPFC and PCC during this task. This finding is interesting given that other studies have found that patients showed hyperconnectivity of these regions during rest in patients with major depression (Wise et al., 2017). The present findings taken together with similar studies during resting state (Wise et al., 2017) and during face processing (King et al., 2023) might further indicate atypical connectivity of the DMN in patients, again potentially suggesting reduced ability to understand others mental states during a face processing task.

When examining the left LP seed, we found statistically significant differences between groups in regions encompassing (1) LLP and bilateral occipital cortex and precuneus, (2) cingulate gyrus and precuneus, (3) left supramarginal gyrus, and (4) a cluster encompassing another region of the precuneus, confirming previously reported findings (Aas et al., 2016; Dauvermann et al., 2021). The precuneus is a brain region involved in a variety of complex functions, which include recollection and memory, integration of information (gestalt) relating to perception of the environment, cue reactivity, mental imagery strategies, episodic memory retrieval, and affective responses to pain. It has been implicated in social cognitive processes in recent research (Bradley et al., 2019). Several studies have shown that, despite being a core component of the DMN (Utevsky, Smith, & Huettel, 2014), precuneus activation increases during tasks such as memory retrieval (Fletcher et al., 1995; Lundstrom, Ingvar, & Petersson, 2005; Maddock, Garrett, & Buonocore, 2001), reward monitoring (Hayden, Nair, McCoy, & Platt, 2008), and emotion processing (Maddock, Garrett, & Buonocore, 2003); see (Cavanna & Trimble, 2006) for review). In the present study, patients have reduced connectivity between the LLP of the DMN and precuneus, indicating impaired emotional processing, during face processing.

For the right LP seed, statistically significant functional connectivity differences between patients and controls were also observed in regions encompassing (1) right occipital cortex and right temporal gyrus (note precuneus was included in this cluster also – See Supplementary Table 2), (2) the left angular gyrus, occipital cortex and precuneus, (3) the cingulate gyrus, precuneus and thalamus, and (4) the precuneus. Functional deficits in these cognitive processes (Onitsuka et al., 2004) have been well documented in patients with schizophrenia and other conditions and the present study demonstrates weaker connections of the DMN RLP seed with these regions and the precuneus, which together might reflect difficulties in accurately perceiving, processing and interpreting visual information of emotional content such as viewing faces.

Finally, functional connectivity differences were also observed between the PCC seed and a large cluster encompassing the precuneus and other brain regions in patients compared to controls. A smaller cluster involving the right frontal gyrus was also observed in patients compared to controls. The precuneus is a medial parietal cortex region implicated in the sense of self, autobiographical memory, and spatial function. The reduced functional connectivity of the DMN PCC seed with the precuneus during face processing suggests atypical precuneus connectivity (in patients) during social cognitive processes. This finding aligns with our previous work (Dauvermann et al., 2021; King et al., 2021) and others, where we previously documented mediating associations between childhood trauma involving DMN dysconnectivity between bilateral cortices and precuneus. Taken together, the findings of this study demonstrate a potential crucial importance of the connectivity between the DMN and precuneus during face processing, which highlight that greater reduction in connections between the DMN and precuneus results in impaired emotion recognition.

The reduced functional connectivity of the DMN with precuneus during face processing was found to mediate the association between a past history of childhood trauma and spatial working memory deficits. This suggests a possible neurodevelopmental process involving emotion processing that in turn, impacts on higher order cognitive processes such as spatial working memory. In order to examine whether this finding was driven by a particular patient group, we found that patients with MDD statistically moderated the mediating effects observed, indicating even greater reduction in DMN connectivity and stronger mediating effects between CT and spatial working memory. The mediating role of low functional connectivity of the DMN (between CT and SWM) with precuneus suggests that childhood traumatic events are associated with a reduced or low sense of self (Wan, Rolls, Feng, & Cheng, 2022). In addition, a low functional connectivity of the precuneus with some cortical areas has previously been shown to be associated with depression (Cheng et al., 2018). The lower connectivity of the precuneus reported here may therefore be related to depression, and contribute particularly to low self-esteem in depression. Although speculative, the lower connectivity of the precuneus may be associated with (e.g., an atypical consequence of) the hyperconnectivity of the mPFC-PCC DMN findings described above, which relate to depression related ruminative cognitions, but this would need to be tested. Therefore, a future study could assess the hypothesis that the mechanism via which CT impacts SWM may be caused by hyperconnectivity of specific regions of the DMN which in turn leads to an instability of the DMN network (i.e., preventing normal/typical activation of DMN), and hence, poor cognitive performance. The idea surrounding ‘instability of the DMN’ was first proposed by Wise and colleagues, see here for their work (Wise et al., 2017). The findings in this study therefore provide evidence of a potential known network (i.e., the DMN) that is involved in the mechanism linking CT with cognitive performance and may at least in part explain the findings in a recent similar study, published last year (Wan et al., 2022). In conclusion, these findings have important implications for treatment and prevention, and it appears critical to consider the impact of early life stress on specific areas of the brain as related to mental health, and in particular, with regards to atypical DMN connectivity during emotion processing and its impact on cognitive impairment.

Mediation was tested statistically, but causality cannot be definitively established. It is assumed that CT (which occurred earlier in life) influenced brain connectivity and then cognition. Longitudinal studies are needed to map the effect of CT on DMN. In the meantime, causal statistics and machine learning methods can be used to further help with establishing causality.

The CTQ demonstrates good to excellent test-retest reliability (Bernstein and fink, 1998) and is widely used, despite the acknowledged issues of recollection bias and subjective self-report in retrospective measures of CT. We discuss important points to consider in (Dauvermann et al., 2019), where researchers have demonstrated correlations between retrospective and prospective recall of CT. Nevertheless, more studies are needed to confirm observations found here.

This study measured Default Mode Network (DMN) connectivity during a social cognitive task. A direct comparison with DMN connectivity during resting-state conditions was not possible due to the unavailability of resting-state data. Future research should aim to include such comparisons to provide a more comprehensive understanding of task-related DMN modulation. The study did not include any direct measures of social cognitive performance and it will be important to include such measures in future studies.

The patient sample was heterogeneous and certain factors could be potential confounds of the data, such as varied psychotropic use and/or clinical status. It will be important in future studies to control for these important confounds.

## Supplementary Material

Supplementary Table

## Data Availability

De-identified data of the IMAGEN, STRATIFY and ESTRA studies are available to researchers after approval of a proposal by the Executive Committee of these studies, chaired by the Centre for Population Neuroscience and Precision Medicine (PONS)s, Charité - University Medicine Berlin (Email: ponscentre@charite.de). The study protocols are available on https://imagen-project.org/ and https://stratify-project.org/. The STRATIFY and ESTRA studies adopt the same study protocols except for the inclusion criteria for different patient groups. The data that support the findings of this study are available on request from the corresponding author. The data are not publicly available due to privacy or ethical restrictions.

## Code availability

The analytic codes are available in the attached ‘custom code’ file and further, upon request from the corresponding author.
