# Supplementary material for: Characterising a stress-sensitive default mode network (DMN) deficit in major psychiatric disorders
Source: Commun Biol. 2026 Feb 25;9:603. doi: 10.1038/s42003-025-09400-1 (PMC7619006; doi:10.1038/s42003-025-09400-1)
Supplement: Supplementary file 2 — Description of Additional Supplementary Materials [file 42003_2025_9400_MOESM2_ESM.pdf]

Description of Additional Supplementary Files

File name: Custom Code

Description: the analytic code
