# Supplementary material for: Characterising a stress-sensitive default mode network (DMN) deficit in major psychiatric disorders
Source: Commun Biol. 2026 Feb 25;9:603. doi: 10.1038/s42003-025-09400-1 (PMC7619006; doi:10.1038/s42003-025-09400-1)
Supplement: Supplementary file 4 — Reporting Summary [file 42003_2025_9400_MOESM4_ESM.pdf]

nature portfolio

Corresponding author(s): *Double-anonymous peer review submissions: write DAPR and your manuscript number here instead of author names.*

Last updated by author(s): *YYYY-MM-DD*

Reporting Summary

Nature Portfolio wishes to improve the reproducibility of the work that we publish. This form provides structure for consistency and transparency in reporting. For further information on Nature Portfolio policies, see our Editorial Policies and the Editorial Policy Checklist.

Statistics

For all statistical analyses, confirm that the following items are present in the figure legend, table legend, main text, or Methods section.

n/a	Confirmed	
☐	☒	The exact sample size (*n*) for each experimental group/condition, given as a discrete number and unit of measurement
☐	☒	A statement on whether measurements were taken from distinct samples or whether the same sample was measured repeatedly
☐	☒	The statistical test(s) used AND whether they are one- or two-sided<br>*Only common tests should be described solely by name; describe more complex techniques in the Methods section.*
☐	☒	A description of all covariates tested
☐	☒	A description of any assumptions or corrections, such as tests of normality and adjustment for multiple comparisons
☐	☒	A full description of the statistical parameters including central tendency (e.g. means) or other basic estimates (e.g. regression coefficient) AND variation (e.g. standard deviation) or associated estimates of uncertainty (e.g. confidence intervals)
☐	☒	For null hypothesis testing, the test statistic (e.g. *F*, *t*, *r*) with confidence intervals, effect sizes, degrees of freedom and *P* value noted<br>*Give P values as exact values whenever suitable.*
☐	☒	For Bayesian analysis, information on the choice of priors and Markov chain Monte Carlo settings
☐	☒	For hierarchical and complex designs, identification of the appropriate level for tests and full reporting of outcomes
☐	☒	Estimates of effect sizes (e.g. Cohen's *d*, Pearson's *r*), indicating how they were calculated

Our web collection on statistics for biologists contains articles on many of the points above.

Software and code

Policy information about availability of computer code

Data collection	no software was used
Data analysis	R 4.5.1 was used to analyse the data in this study

For manuscripts utilizing custom algorithms or software that are central to the research but not yet described in published literature, software must be made available to editors and reviewers. We strongly encourage code deposition in a community repository (e.g. GitHub). See the Nature Portfolio guidelines for submitting code & software for further information.

Data

Policy information about availability of data

All manuscripts must include a data availability statement. This statement should provide the following information, where applicable:

- Accession codes, unique identifiers, or web links for publicly available datasets
- A description of any restrictions on data availability
- For clinical datasets or third party data, please ensure that the statement adheres to our policy

De-identified data of the IMAGEN, STRATIFY and ESTRA studies are available to researchers after approval of a proposal by the Executive Committee of these studies, chaired by the Centre for Population Neuroscience and Precision Medicine (PONS)s, Charité - University Medicine Berlin (Email: ponscentre@charite.de). The study protocols are available on https://imagen-project.org/ and https://stratify-project.org/. The STRATIFY and ESTRA studies adopt the same study protocols

except for the inclusion criteria for different patient groups. The analytic codes are available on request from the corresponding author.

Research involving human participants, their data, or biological material

Policy information about studies with human participants or human data. See also policy information about sex, gender (identity/presentation), and sexual orientation and race, ethnicity and racism.

Reporting on sex and gender	Sex data is analysed and reported throughout the manuscript. Gender data is not available and therefore not presented.
Reporting on race, ethnicity, or other socially relevant groupings	The sample included white participants only and is described as such.
Population characteristics	See above
Recruitment	Participants were recruited from improving access to psychological therapy services and other relevant clinics as described and outline in the manuscript. No self selection bias could have been made.
Ethics oversight	All procedures involving human subjects/patients were approved by King's College London Research Ethics Committee (17/LO/0552) for IMAGEN, London Westminster Research Ethics Committee (PNM/10/11-126) for STRATIFY and North West–Greater Manchester South Research Ethics Committee (20/NW/0143) for ESTRA.

Note that full information on the approval of the study protocol must also be provided in the manuscript.

Field-specific reporting

Please select the one below that is the best fit for your research. If you are not sure, read the appropriate sections before making your selection.

☐ Life sciences ☒ Behavioural & social sciences ☐ Ecological, evolutionary & environmental sciences

For a reference copy of the document with all sections, see nature.com/documents/nr-reporting-summary-flat.pdf

Behavioural & social sciences study design

All studies must disclose on these points even when the disclosure is negative.

Study description	quantitative cross sectional
Research sample	Young adults recruited from IMAGEN and STRATIFY cohorts
Sampling strategy	All available and eligible data were included in the current study, from both IMAGEN, STRATIFY and ESTRA cohorts.
Data collection	A mixture between pen and paper (e.g., MINI) and computer (e.g., CANTAB tasks) were used
Timing	Data were collected over one study visit on one day over the course of 4-5 hours.
Data exclusions	No data were excluded from the analyses
Non-participation	This is a secondary data analysis of already collected data therefore no particpants dropped out.
Randomization	particpants were not randomly allocated.

Reporting for specific materials, systems and methods

We require information from authors about some types of materials, experimental systems and methods used in many studies. Here, indicate whether each material, system or method listed is relevant to your study. If you are not sure if a list item applies to your research, read the appropriate section before selecting a response.

Materials & experimental systems

n/a	Involved in the study
☒	☐ Antibodies
☒	☐ Eukaryotic cell lines
☒	☐ Palaeontology and archaeology
☒	☐ Animals and other organisms
☒	☐ Clinical data
☒	☐ Dual use research of concern
☒	☐ Plants

Methods

n/a	Involved in the study
☒	☐ ChIP-seq
☒	☐ Flow cytometry
☐	☒ MRI-based neuroimaging

Plants

Seed stocks: na

Novel plant genotypes: na

Authentication: na

Magnetic resonance imaging

Experimental design

Design type: resting state during task performance

Design specifications: 18-second blocks of either a face movie (depicting anger or neutrality) or a control stimulus. Each face movie showed black and white video clips (200–500ms) of male or female faces. Five blocks each of angry and neutral expressions were interleaved with nine blocks of the control stimulus. Each block contained eight trials of six face identities (three female).

Behavioral performance measures: passive viewing

Acquisition

Imaging type(s): functional

Field strength: 3T

Sequence & imaging parameters: EPI

Area of acquisition: Whole brain

Diffusion MRI: ☐ Used ☒ Not used

Preprocessing

Preprocessing software: CONN Toolbox

Normalization: nonlinearly warping to the MNI space (on the basis of a custom echo-planar imaging template (53×63×46voxels) created from an average of the mean images from 400 adolescents), resampling at a resolution of 3×3×3mm3 and smoothing with an isotropic Gaussian kernel of 5mm full-width at half-maximum.

Normalization template: nonlinearly warping to the MNI space (on the basis of a custom echo-planar imaging template (53×63×46voxels) created from an average of the mean images from 400 adolescents), resampling at a resolution of 3×3×3mm3 and smoothing with an isotropic Gaussian kernel of 5mm full-width at half-maximum.

Noise and artifact removal: nonlinearly warping to the MNI space (on the basis of a custom echo-planar imaging template (53×63×46voxels) created from an average of the mean images from 400 adolescents), resampling at a resolution of 3×3×3mm3 and smoothing with an isotropic Gaussian kernel of 5mm full-width at half-maximum.

Volume censoring	Further preprocessing and QC measures were carried out as described elsewhere (King et al., 2021). Briefly, scans underwent 'motion scrubbing' and anatomical component-based noise correction (aCompCor) in CONN, employing structural white matter (WM) and cerebrospinal fluid (CSF) masks to generate effective motion-reducing regressors. Additional head motion variance was addressed by including regressors from the 6 motion correction parameters and their temporal derivatives (GM, WM, CSF) during band-pass filtering (.01–.10 Hz).

Statistical modeling & inference

Model type and settings	univariate mediation modelling were used
Effect(s) tested	t test

Specify type of analysis: ☒ Whole brain ☐ ROI-based ☐ Both

Statistic type for inference (See Eklund et al. 2016)	FWE <.001 cluster level
Correction	FWE was used using the threshold of <.001

Models & analysis

n/a	Involved in the study
☐	☒ Functional and/or effective connectivity
☒	☐ Graph analysis
☒	☐ Multivariate modeling or predictive analysis

Functional and/or effective connectivity	t test
