# Supplementary material for: Characterising a stress-sensitive default mode network (DMN) deficit in major psychiatric disorders
Source: Commun Biol. Author manuscript; Available in PMC 2026 Apr 15. (PMC7619006; doi:10.1038/s42003-025-09400-1)
Supplement: Supplementary file 1 [file EMS213144-supplement-Supplementary_Table.pdf]

Supplementary Table 1 - Demographic, Clinical, Environmental, and Neuropsychological Data

	**Clinical sample (N=433)**	**Controls (N=1418)**							**Major Depression (N=135)**		**Eating Disorders (N=127)**		**Alcohol Use Disorder (N=108)**		**Psychosis** (N=58)	
	M (SD)/%	(M (SD)/%	p-value	x^2/t-value	*d*	*CI low*	*CI high*	df	M (SD)/%	*Cohens d [CI Low, CI High]*	M (SD)/%	*Cohens d [CI Low, CI High]*	M (SD)/%	*Cohens d [CI Low, CI High]*	M (SD)/%	*Cohens d [CI Low, CI High]*
**Demographics**																
Sex	58% female	56% female	0.15	.81					54% female		100% female		52% female		51% female	
Age	21.7 (2.1)	21.9 (1.5)	0.3	1.5					22.1 (1.6)		21.8 (1.8)		20.8 (2.1)		21.5 (1.9)	
Verbal IQ	109 (1.1)	110 (1.2)	0.1	1.1												
Handedness (Right)	304 (70.2%)	1030 (72.6%)														
**Childhood Trauma Questionnaire (CTQ)**																
CTQ Total Scores	15.1 (13.8)	7.3 (8.5)	<.001	10.4	*0.64*	*0.54*	*0.73*	1795	16.3 (13.5)	*.45 [.36, .55]*	16.5 (14.3)	*.46 [.35, .54]*	14.1 (14.1)	*.38 [.29, .48]*	15.5 (13.8)	*.09 [-.001, .18]*
Emotional Abuse	4.7 (4.4)	2.2 (2.9)	<.001	10.3	*0.61*	*0.514*	*0.703*	1795	4.7 (4.1)	*.41 [.31, .5]*	5.2 (4.7)	*.43 [.33, .52]*	4.7 (4.7)	*.4 [.3, .49]*	4.8 (4.7)	*.05 [-.04, .14]*
Physical Abuse	1 (2.2)	.47 (1.4)	<.001	3.4	*0.21*	*0.115*	*0.301*	1795	.9 (2.3)	*.14 [.05, .24]*	1.2 (2.7)	*.19 [.09, .28]*	.76 (1.8)	*.09 [0, .19]*	.5 (1.2)	*.03 [-.13, .06]*
Sexual Abuse	1.6 (3.3)	.39 (1.9)	<.001	6.8	*0.44*	*0.348*	*0.535*	1793	1.5 (2.8)	*.29 [.19, .38]*	1.7 (3.1)	*.29 [.2, .39]*	1.5 (3.7)	*.28 [.19, .38]*	1.5 (3.4)	*.16 [.06, .25]*
Emotional Neglect	5.3 (4.6)	3.1 (3.2)	<.001	8.8	*0.51*	*0.413*	*0.601*	1795	5.7 (4.6)	*.4 [.3, .49]*	5.6 (4.7)	*.34 [.24, .43]*	4.8 (4.4)	*.26 [.17 .36]*	5.1 (4.9)	*.05 [-.04, .15]*
Physical Neglect	2.4 (3.1)	1 (1.8)	<.001	8.3	*0.52*	*0.425*	*0.613*	1795	2.3 (3.2)	*.35 [.25, .44]*	2.6 (3.1)	*.37 [.27, .46]*	2.3 (2.9)	*.31 [.21, .4]*	2.4 (3.0)	*0.11 [.02, .21]*
**Neuropsychological Assessment**																

Cambridge Gambling Task (CGT)																
CGT Delay Aversion	.22 (.21)	.17 (.14)			*0.26*	*0.161*	*0.351*	1719	.22 (.18)	*.17 [.07, .26]*	.26 (.22)	*.05 [-.05, .14]*	.2 (.24)	*.26 [.17, .36]*	.2 (.21)	*.08 [-.02, .17]*
CGT Deliberation Time	1605.6 (523.8)	1709.2 (4961.2)	<.001	4.3	*-0.03*	*-0.12*	*0.068*	1719	1647.9 (547.6)	*-.02 [-.11, .08]*	1512.0 (303.2)	*.02 [-.11, .08]*	1573 (477.5)	*.02 [-.11, .08]*	1525 (468.2)	*0 [-.1, .09]*
CGT Risk Adjustment	1.5 (.98)	1.9 (1.0)	<.001	-7.1	*-0.34*	*-0.435*	*-0.244*	1719	1.6 (.96)	*.19 [.07, .26]*	1.4 (.98)	*.05 [-.05, .14]*	1.7 (1.0)	*.26 [.17, .36]*	1.5 (.90)	*.08 [-.02, .17]*
Intra-Extra Dimensional Shift (IED)																
IED Total Trials	80.6 (19.3)	77.6 (17.2)	0.005	2.8	*0.14*	*0.049*	*0.239*	1719	82.9 (20.1)	*.17 [.07, .27]*	81.3 (19.9)	*.02 [-.12, .08]*	76.9 (16.9)	*.1 [.01, .2]*	78.5 (18.8)	*.06 [-.04, .15]*
IED Total Trials Adjusted	89.9 (37.0)	86.2 (36.9)	0.081	1.7	*0.08*	*0.01*	*0.17*	1719	94.5 (35.3)	*.14 [.03, .24]*	92.4 (46.5)	*.12 [.05, .15]*	81.6 (26.6)	*.09 [0, .15]*	85.6 (25.2)	*.06 [.01, .11]*
IED Total Errors	17.1 (11.6)	15.6 (10.3)	/021	2.2	*-0.12*	*0.02*	*0.21*	1719	18.63 (12.2)	*.18 [.07, .28]*	17.8 (12.9)	*.16 [.1, .23]*	14.6 (9.6)	*.05 [.01, .11]*	16.5 (10.5)	*.13 [.05, .19]*
IED Stages completed	8.6 (.91)	8.7 (.86)	0.13	-.78	*-0.04*	*-0.13*	*0.05*	1719	8.49 (.84)	*-.11 [-.21, -.007]*	8.6 (.98)	*.11 [.01, .19]*	8.8 (.59)	*.13 [.06, .22]*	8.5 (.76)	*-.01 [-.04 [0, .18]*
Spatial Working Memory (SWM)																
Between Errors	19.69 (17.3)	12.7 (12.8)	<.001	7.3	*0.42*	*0.321*	*0.512*	1719	20.4 (16.6)	*.32 [.22, .42]*	22.8 (19.9)	*.11 [0, .19]*	15.2 (14.2)	*.36 [.27, .46]*	19.5 (16.5)	*.12 [.02, .21]*
SWM Strategy	30.8 (6.2)	27.8 (6.2)	<.001	8.3	*0.4*	*0.305*	*0.496*	1719	30.8 (5.8)	*.28 [.19, .38]*	31.8 (6.7)	*.32 [.22, .41]*	29.8 (6.0)	*.18 [.08, .27]*	30.1 (6.1)	*.07 [-.03, .17]*

IED set shift test: number of stages completed = a higher score is better; errors = a lower score is better; SWM errors scale (i.e., a higher score means more errors) and SWM strategy (i.e., a higher score indicates a less effective strategy); d = Cohens d statistic; CI = CI low, CI high

Supplementary Table 2 – Comparisons in DMN connectivity between patients and controls

	MNI			Voxels	Peak T	Height threshold *P* value uncorrected
	x	y	z			
**Medial PFC Seed**						
1756 voxels (24%) covering 25% of atlas.FP l (Frontal Pole Left)<br>1418 voxels (20%) covering 18% of atlas.FP r (Frontal Pole Right)<br>686 voxels (10%) covering 26% of atlas.AC (Cingulate Gyrus, anterior division)<br>626 voxels (9%) covering 46% of atlas.PaCiG r (Paracingulate Gyrus Right)<br>625 voxels (9%) covering 48% of atlas.PaCiG l (Paracingulate Gyrus Left)<br>330 voxels (5%) covering 34% of atlas.MedFC (Frontal Medial Cortex)<br>94 voxels (1%) covering 3% of atlas.SFG l (Superior Frontal Gyrus Left)<br>79 voxels (1%) covering 3% of atlas.SFG r (Superior Frontal Gyrus Right)<br>56 voxels (1%) covering 5% of atlas.SubCalC (Subcallosal Cortex)<br>40 voxels (1%) covering 2% of atlas.FOrb l (Frontal Orbital Cortex Left)<br>1464 voxels (20%) covering 0% of atlas.not-labeled	+00	+56	+00	7174		<.001 (FWE)
**MPFC**						
277 voxels (35%) covering 12% of atlas.PC (Cingulate Gyrus, posterior division)<br>40 voxels (5%) covering 3% of atlas.Thalamus l<br>9 voxels (1%) covering 0% of atlas.AC (Cingulate Gyrus, anterior division)<br>4 voxels (1%) covering 0% of atlas.Thalamus r<br>468 voxels (59%) covering 0% of atlas.not-labeled	-04	-22	+22	798		<.001(FWE)
**MPFC**						
205 voxels (52%) covering 9% of atlas.TP l (Temporal Pole Left)<br><br>48 voxels (12%) covering 17% of atlas.aSTG l (Superior Temporal Gyrus, anterior division Left)<br><br>20 voxels (5%) covering 3% of atlas.IFG oper l (Inferior Frontal Gyrus, pars opercularis Left)	-56	+16	-18	394		<.001 (FWE)

16 voxels (4%) covering 4% of atlas.aMTG l (Middle Temporal Gyrus, anterior division Left)<br><br>15 voxels (4%) covering 1% of atlas.FOrb l (Frontal Orbital Cortex Left)<br><br>9 voxels (2%) covering 1% of atlas.IFG tri l (Inferior Frontal Gyrus, pars triangularis Left)<br><br>7 voxels (2%) covering 2% of atlas.PP l (Planum Polare Left)<br><br>5 voxels (1%) covering 0% of atlas.PreCG l (Precentral Gyrus Left)<br><br>3 voxels (1%) covering 1% of atlas.FO l (Frontal Operculum Cortex Left)<br><br>66 voxels (17%) covering 0% of atlas.not-labeled						
**MPFC**						
160 voxels (45%) covering 7% of atlas.TP r (Temporal Pole Right)<br>33 voxels (9%) covering 2% of atlas.FOrb r (Frontal Orbital Cortex Right)<br>23 voxels (6%) covering 8% of atlas.aSTG r (Superior Temporal Gyrus, anterior division Right)<br>11 voxels (3%) covering 2% of atlas.IFG oper r (Inferior Frontal Gyrus, pars opercularis Right)<br>7 voxels (2%) covering 1% of atlas.IFG tri r (Inferior Frontal Gyrus, pars triangularis Right)<br>7 voxels (2%) covering 2% of atlas.aMTG r (Middle Temporal Gyrus, anterior division Right)<br>4 voxels (1%) covering 0% of atlas.IC r (Insular Cortex Right)<br>2 voxels (1%) covering 0% of atlas.PreCG r (Precentral Gyrus Right)<br>1 voxels (0%) covering 0% of atlas.FP r (Frontal Pole Right)<br>106 voxels (30%) covering 0% of atlas.not-labeled	+54	+14	-.04			<.001 (FWE)

**Brain Area**	**MNI**			**Voxels**	**Peak T**	**Height threshold *P* value uncorrected**
	**x**	**y**	**z**			

**LLP Seed**						
2728 voxels (46%) covering 55% of atlas.sLOC l (Lateral Occipital Cortex, superior division Left)<br>524 voxels (9%) covering 55% of atlas.AG l (Angular Gyrus Left)<br>468 voxels (8%) covering 10% of atlas.sLOC r (Lateral Occipital Cortex, superior division Right)<br>307 voxels (5%) covering 29% of atlas.pSMG l (Supramarginal Gyrus, posterior division Left)<br>192 voxels (3%) covering 3% of atlas.Precuneous (Precuneous Cortex)<br>111 voxels (2%) covering 21% of atlas.Cuneal l (Cuneal Cortex Left)<br>105 voxels (2%) covering 3% of atlas.PostCG l (Postcentral Gyrus Left)<br>105 voxels (2%) covering 11% of atlas.aSMG l (Supramarginal Gyrus, anterior division Left)<br>93 voxels (2%) covering 4% of atlas.OP l (Occipital Pole Left)<br>82 voxels (1%) covering 3% of atlas.PC (Cingulate Gyrus, posterior division)<br>73 voxels (1%) covering 4% of atlas.iLOC l (Lateral Occipital Cortex, inferior division Left)<br>66 voxels (1%) covering 8% of atlas.toMTG l (Middle Temporal Gyrus, temporooccipital part Left)<br>58 voxels (1%) covering 4% of atlas.SPL l (Superior Parietal Lobule Left)<br>43 voxels (1%) covering 1% of atlas.PreCG l (Precentral Gyrus Left)<br>38 voxels (1%) covering 7% of atlas.PO l (Parietal Operculum Cortex Left)<br>38 voxels (1%) covering 7% of atlas.PT l (Planum Temporale Left)<br>26 voxels (0%) covering 4% of atlas.Cuneal r (Cuneal Cortex Right)<br>25 voxels (0%) covering 1% of atlas.OP r (Occipital Pole Right)<br>19 voxels (0%) covering 1% of atlas.pMTG l (Middle Temporal Gyrus, posterior division Left)<br>8 voxels (0%) covering 1% of atlas.IFG oper l (Inferior Frontal Gyrus, pars opercularis Left)<br>7 voxels (0%) covering 0% of atlas.AG r (Angular Gyrus Right)<br>6 voxels (0%) covering 2% of atlas.aSTG l (Superior Temporal Gyrus, anterior division Left)<br>3 voxels (0%) covering 1% of atlas.pSTG l (Superior Temporal Gyrus, posterior division Left)<br>2 voxels (0%) covering 0% of atlas.Ver45 (Vermis 4 5)	-40	-68	+24	5688		<.001 (FWE)

856 voxels (14%) covering 0% of atlas.not-labeled						
**LLP Seed**						
69 voxels (74%) covering 3% of atlas.PC (Cingulate Gyrus, posterior division)<br>24 voxels (26%) covering 0% of atlas.Precuneous (Precuneous Cortex)	-10	-48	+14	93		<.001(FWE)
**LLP Seed**						
18 voxels (40%) covering 2% of atlas.pSMG l (Supramarginal Gyrus, posterior division Left)<br><br>12 voxels (27%) covering 1% of atlas.aSMG l (Supramarginal Gyrus, anterior division Left)<br><br>8 voxels (18%) covering 0% of atlas.PostCG l (Postcentral Gyrus Left)<br><br>7 voxels (16%) covering 0% of atlas.not-labeled	-56	-42	+52	45		<.001 (FWE)
**LLP Seed**						
19 voxels (86%) covering atlas.Precuneous (Precuneous Cortex)<br>3 voxels (14%) covering 0% of atlas.not-labeled	-10	-58	+46	22		<.001 (FWE)
**Brain Area**	**MNI**			**Voxels**	**Peak T**	**Height threshold *P* value uncorrected**
	**x**	**y**	**z**			
**RLP Seed**						
1649 voxels (32%) covering 34% of atlas.sLOC r (Lateral Occipital Cortex, superior division Right)<br>777 voxels (15%) covering 53% of atlas.AG r (Angular Gyrus Right)<br>397 voxels (8%) covering 32% of atlas.pSMG r (Supramarginal Gyrus, posterior division Right)<br>226 voxels (4%) covering 19% of atlas.toMTG r (Middle Temporal Gyrus, temporooccipital part Right)<br>180 voxels (4%) covering 22% of atlas.aSMG r (Supramarginal Gyrus, anterior division Right)	-46	-54	+20	5089		<.001 (FWE)

115 voxels (2%) covering 6% of atlas.iLOC r (Lateral Occipital Cortex, inferior division Right)<br>98 voxels (2%) covering 3% of atlas.PostCG r (Postcentral Gyrus Right)<br>78 voxels (2%) covering 14% of atlas.PO r (Parietal Operculum Cortex Right)<br>40 voxels (1%) covering 6% of atlas.Cuneal r (Cuneal Cortex Right)<br>35 voxels (1%) covering 8% of atlas.PT r (Planum Temporale Right)<br>33 voxels (1%) covering 2% of atlas.SPL r (Superior Parietal Lobule Right)<br>24 voxels (0%) covering 0% of atlas.Precuneous (Precuneous Cortex)<br>21 voxels (0%) covering 5% of atlas.pSTG r (Superior Temporal Gyrus, posterior division Right)<br>14 voxels (0%) covering 1% of atlas.pMTG r (Middle Temporal Gyrus, posterior division Right)<br>9 voxels (0%) covering 1% of atlas.ICC r (Intracalcarine Cortex Right)<br>7 voxels (0%) covering 1% of atlas.CO r (Central Opercular Cortex Right)<br>1386 voxels (27%) covering 0% of atlas.not-labeled						
**RLP Seed**						
98 voxels (13%) covering 10% of atlas.AG l (Angular Gyrus Left)<br>24 voxels (3%) covering 5% of atlas.Cuneal l (Cuneal Cortex Left)<br>4 voxels (1%) covering 0% of atlas.Precuneous (Precuneous Cortex)<br>1 voxels (0%) covering 0% of atlas.pSMG l (Supramarginal Gyrus, posterior division Left)<br>1 voxels (0%) covering 0% of atlas.iLOC l (Lateral Occipital Cortex, inferior division Left)<br>508 voxels (67%) covering 0% of atlas.not-labeled	-34	-58	+24	760		<.001(FWE)
**RLP Seed**						
193 voxels (39%) covering 8% of atlas.PC (Cingulate Gyrus, posterior division)<br>110 voxels (22%) covering 2% of atlas.Precuneous (Precuneous Cortex)<br>14 voxels (3%) covering 3% of atlas.Cuneal l (Cuneal Cortex Left)<br>6 voxels (1%) covering 0% of atlas.Thalamus r	-02	-46	+20	494		<.001 (FWE)

2 voxels (0%) covering 0% of atlas.ICC l (Intracalcarine Cortex Left)<br><br>1 voxels (0%) covering 0% of atlas.Ver45 (Vermis 4 5)<br><br>168 voxels (34%) covering 0% of atlas.not-labeled						
**RLP Seed**						
22 voxels (100%) covering 0% of atlas.Precuneous (Precuneous Cortex)	+00	-66	+36	22		<.001 (FWE)

**Brain Area**	**MNI**			**Voxels**	**Peak T**	**Height threshold *P* value uncorrected**
	**x**	**y**	**z**			
**PCC Seed**						
5412 voxels (15%) covering 96% of atlas.Precuneous (Precuneous Cortex)<br>2302 voxels (6%) covering 96% of atlas.PC (Cingulate Gyrus, posterior division)<br>965 voxels (3%) covering 19% of atlas.sLOC l (Lateral Occipital Cortex, superior division Left)<br>947 voxels (3%) covering 20% of atlas.sLOC r (Lateral Occipital Cortex, superior division Right)<br>903 voxels (2%) covering 52% of atlas.LG r (Lingual Gyrus Right)<br>803 voxels (2%) covering 53% of atlas.LG l (Lingual Gyrus Left)<br>764 voxels (2%) covering 24% of atlas.PostCG r (Postcentral Gyrus Right)<br>749 voxels (2%) covering 51% of atlas.SPL r (Superior Parietal Lobule Right)<br>645 voxels (2%) covering 51% of atlas.Thalamus r<br>629 voxels (2%) covering 46% of atlas.Thalamus l<br>614 voxels (2%) covering 42% of atlas.SPL l (Superior Parietal Lobule Left)<br>583 voxels (2%) covering 93% of atlas.Ver45 (Vermis 4 5)<br>547 voxels (1%) covering 15% of atlas.PostCG l (Postcentral Gyrus Left)<br>522 voxels (1%) covering 69% of atlas.ICC r (Intracalcarine Cortex Right)<br>513 voxels (1%) covering 80% of atlas.ICC l (Intracalcarine Cortex Left)<br>459 voxels (1%) covering 11% of atlas.PreCG r (Precentral Gyrus Right)	-04	-56	+28	36384		<.001 (FWE)

438 voxels (1%) covering 10% of atlas.PreCG l (Precentral Gyrus Left)<br>416 voxels (1%) covering 65% of atlas.Cuneal r (Cuneal Cortex Right)<br>407 voxels (1%) covering 45% of atlas.Cereb45 l (Cerebelum 4 5 Left)<br>348 voxels (1%) covering 24% of atlas.AG r (Angular Gyrus Right)<br>334 voxels (1%) covering 64% of atlas.Cuneal l (Cuneal Cortex Left)<br>281 voxels (1%) covering 23% of atlas.pSMG r (Supramarginal Gyrus, posterior division Right)<br>257 voxels (1%) covering 10% of atlas.AC (Cingulate Gyrus, anterior division)<br>252 voxels (1%) covering 41% of atlas.Cereb45 r (Cerebelum 4 5 Right)<br>190 voxels (1%) covering 57% of atlas.Ver6 (Vermis 6)<br>177 voxels (0%) covering 23% of atlas.Hippocampus l<br>145 voxels (0%) covering 15% of atlas.AG l (Angular Gyrus Left)<br>140 voxels (0%) covering 17% of atlas.aSMG r (Supramarginal Gyrus, anterior division Right)<br>124 voxels (0%) covering 87% of atlas.SCC r (Supracalcarine Cortex Right)<br>124 voxels (0%) covering 3% of atlas.Brain-Stem<br>122 voxels (0%) covering 53% of atlas.Ver3 (Vermis 3)<br>119 voxels (0%) covering 22% of atlas.PO r (Parietal Operculum Cortex Right)<br>107 voxels (0%) covering 15% of atlas.Hippocampus r<br>95 voxels (0%) covering 24% of atlas.pPaHC l (Parahippocampal Gyrus, posterior division Left)<br>89 voxels (0%) covering 7% of atlas.Cereb6 l (Cerebelum 6 Left)<br>75 voxels (0%) covering 3% of atlas.SFG r (Superior Frontal Gyrus Right)<br>69 voxels (0%) covering 4% of atlas.Cereb6 r (Cerebelum 6 Right)<br>65 voxels (0%) covering 89% of atlas.SCC l (Supracalcarine Cortex Left)<br>60 voxels (0%) covering 19% of atlas.pPaHC r (Parahippocampal Gyrus, posterior division Right)<br>56 voxels (0%) covering 5% of atlas.pSMG l (Supramarginal Gyrus, posterior division Left)<br>45 voxels (0%) covering 6% of atlas.SMA r (Juxtapositional Lobule Cortex -formerly Supplementary Motor Cortex- Right)<br>42 voxels (0%) covering 7% of atlas.PT l (Planum Temporale Left)						

37 voxels (0%) covering 4% of atlas.aSMG l (Supramarginal Gyrus, anterior division Left)<br>34 voxels (0%) covering 6% of atlas.PO l (Parietal Operculum Cortex Left)<br>34 voxels (0%) covering 26% of atlas.Cereb3 l (Cerebelum 3 Left)<br>21 voxels (0%) covering 1% of atlas.iLOC r (Lateral Occipital Cortex, inferior division Right)<br>18 voxels (0%) covering 6% of atlas.Pallidum l<br>17 voxels (0%) covering 1% of atlas.toMTG r (Middle Temporal Gyrus, temporooccipital part Right)<br>17 voxels (0%) covering 4% of atlas.PT r (Planum Temporale Right)<br>15 voxels (0%) covering 8% of atlas.Cereb3 r (Cerebelum 3 Right)<br>12 voxels (0%) covering 4% of atlas.Pallidum r<br>11 voxels (0%) covering 2% of atlas.SMA L(Juxtapositional Lobule Cortex -formerly Supplementary Motor Cortex- Left)<br>11 voxels (0%) covering 1% of atlas.TOFusC r (Temporal Occipital Fusiform Cortex Right)<br>11 voxels (0%) covering 1% of atlas.Putamen l<br>10 voxels (0%) covering 0% of atlas.iLOC l (Lateral Occipital Cortex, inferior division Left)<br>8 voxels (0%) covering 3% of atlas.Ver8 (Vermis 8)<br>5 voxels (0%) covering 0% of atlas.MidFG r (Middle Frontal Gyrus Right)<br>5 voxels (0%) covering 3% of atlas.Ver7 (Vermis 7)<br>4 voxels (0%) covering 1% of atlas.TOFusC l (Temporal Occipital Fusiform Cortex Left)<br>3 voxels (0%) covering 0% of atlas.IC l (Insular Cortex Left)<br>3 voxels (0%) covering 0% of atlas.toMTG l (Middle Temporal Gyrus, temporooccipital part Left)<br>1 voxels (0%) covering 0% of atlas.FP l (Frontal Pole Left)<br>1 voxels (0%) covering 0% of atlas.IC r (Insular Cortex Right)<br>1 voxels (0%) covering 0% of atlas.pTFusC r (Temporal Fusiform Cortex, posterior division Right)<br>1 voxels (0%) covering 0% of atlas.Cereb8 r (Cerebelum 8 Right)<br>1 voxels (0%) covering 1% of atlas.Ver9 (Vermis 9)<br>14269 voxels (39%) covering 4% of atlas.not-labeled						
**PCC Seed**						

75 voxels (79%) covering 3% of atlas.SFG r (Superior Frontal Gyrus Right)<br>5 voxels (5%) covering 0% of atlas.MidFG r (Middle Frontal Gyrus Right)<br>15 voxels (16%) covering 0% of atlas.not-labeled	+24	-28	+58	94		<.001(FWE)

Supplementary Table 3 - Differences in DMN connectivity between patient groups and controls

	**coefficient**	**std.error**	**statistic**	**p value**	**MNI Coordinates/ Brain Region**
MDD	-0.004396843	0.007152023	-0.614769078	0.539089236	0560DMNMPFC
ED	0.004488874	0.00718977	0.624341813	0.532792105	0560DMNMPFC
AUD	0.005305518	0.007273984	0.729382738	0.466234082	0560DMNMPFC
Psychosis	-0.001983446	0.015944807	-0.124394481	0.901071233	0560DMNMPFC
MDD	0.015927257	0.013734234	1.159675683	0.24693684	42222DMNMPFC
ED	0.03856173	0.01380672	2.792968284	0.005497095	42222DMNMPFC
AUD	0.029886493	0.013968438	2.139573019	0.033050208	42222DMNMPFC
Psychosis	-0.022625049	0.030619266	-0.738915456	0.46043193	42222DMNMPFC
MDD	-0.001443006	0.009320281	-0.154824293	0.877045183	561618DMNMPFC
ED	-0.005700399	0.009369471	-0.608401416	0.54329865	561618DMNMPFC
AUD	-0.010127524	0.009479216	-1.068392654	0.286047581	561618DMNMPFC
Psychosis	-0.032814882	0.020778746	-1.579252252	0.115142037	561618DMNMPFC
MDD	-0.034919993	0.006992502	-4.993919273	9.18E-07	406824DMNLPl
ED	-0.047901759	0.007029407	-6.814480602	3.91E-11	406824DMNLPl
AUD	-0.029793973	0.007111743	-4.189405357	3.51E-05	406824DMNLPl
Psychosis	-0.009571317	0.015589169	-0.613972234	0.539615099	406824DMNLPl
MDD	-0.029550014	0.012200456	-2.422041698	0.015918674	104814DMNLPl
ED	-0.035480152	0.012264847	-2.892832802	0.004046189	104814DMNLPl
AUD	-0.032005361	0.012408506	-2.579308253	0.010289351	104814DMNLPl
Psychosis	-0.050995617	0.027199844	-1.874849613	0.061609564	104814DMNLPl
MDD	-0.050812417	0.011790386	-4.309648306	2.10E-05	564252DMNLPl
ED	-0.053381608	0.011852613	-4.50378396	8.99E-06	564252DMNLPl
AUD	-0.040805932	0.011991443	-3.402921013	0.000740421	564252DMNLPl
Psychosis	-0.082057178	0.026285629	-3.12175064	0.001940756	564252DMNLPl
MDD	-0.026315665	0.008366969	-3.145185059	0.001795597	465420DMNLPr
ED	-0.044177117	0.008411128	-5.252223001	2.56E-07	465420DMNLPr
AUD	-0.014910386	0.008509647	-1.7521744	0.080581245	465420DMNLPr
Psychosis	-0.010018575	0.018653421	-0.537090493	0.591531462	465420DMNLPr
MDD	-0.022965117	0.011408688	-2.01294976	0.044851751	345824DMNLPr
ED	-0.035229889	0.011468901	-3.071775525	0.002287124	345824DMNLPr
AUD	-0.012499261	0.011603236	-1.077222012	0.282090591	345824DMNLPr
Psychosis	-0.033066007	0.025434667	-1.300036928	0.194406421	345824DMNLPr
MDD	-0.019663788	0.010550157	-1.863838519	0.063144908	24620DMNLPr
ED	-0.031051448	0.010605838	-2.927769432	0.003627522	24620DMNLPr
AUD	-0.020092994	0.010730064	-1.872588464	0.061922292	24620DMNLPr
Psychosis	-0.028381105	0.023520646	-1.206646456	0.228347658	24620DMNLPr
MDD	-0.005749183	0.0056405	-1.019268476	0.857395646	45628DMNPCC
ED	0.000368373	0.005670269	0.064965684	0.519510162	45628DMNPCC
AUD	0.000192427	0.005736685	0.033543167	0.410097963	45628DMNPCC
Psychosis	-0.033748718	0.012574998	-2.683795164	0.154643309	45628DMNPCC
MDD	0.00213503	0.011873339	0.179817132	0.556510822	242258DMNPCC
ED	-0.007695424	0.011936004	-0.64472367	0.34017178	242258DMNPCC

AUD	-0.009958519	0.01207581	-0.824666703	0.18200259	242258DMNPCC
Psychosis	-0.037754129	0.026470566	-1.426268316	0.485166447	242258DMNPCC

Note: MDD=Major Depression, ED= Eating Disorders, AUD= Alcohol Use Disorders, MNI=Montreal Neurological Institute, coefficient = a representation of the mean difference in connectivity between each patient group and controls.
